# Does hypothyroidism increase the risk of breast cancer: evidence from a meta-analysis

**DOI:** 10.1186/s12885-020-07230-4

**Published:** 2020-08-06

**Authors:** Bolin Wang, Zhong Lu, Yan Huang, Ruobao Li, Tao Lin

**Affiliations:** 1 School of Clinical Medicine, Weifang Medical University, Weifang, 261053 China; 2 Department of Oncology, Affiliated Hospital of Weifang Medical University, Weifang, 261031 China; 3 School of Basic Medicine, Weifang Medical University, Weifang, 261053 China

**Keywords:** Hypothyroidism, Thyroid hormone replacement therapy, Breast cancer, Meta-analysis

## Abstract

**Purpose:**

At present, the relationship between hypothyroidism and the risk of breast cancer is still inconclusive. This meta-analysis was used to systematically assess the relationship between hypothyroidism and breast cancer risk, and to assess whether thyroid hormone replacement therapy can increase breast cancer risk.

**Methods:**

The relevant articles about hypothyroidism and the risk of breast cancer were obtained on the electronic database platform. Relevant data were extracted, and odd ratios (OR) with corresponding 95% confidence intervals (CI) were merged using Stata SE 12.0 software.

**Results:**

A total of 19 related studies were included in the meta-analysis, including 6 cohort studies and 13 case-control studies. The results show that hypothyroidism was not related to the risk of breast cancer (odd ratios = 0.90, 95% CI 0.77–1.03). In the European subgroup, we observed that patients with hypothyroidism have a lower risk of breast cancer(odd ratios = 0.93, 95% CI 0.88–0.99). Furthermore, no significant correlation was observed between thyroid hormone replacement therapy and the risk of breast cancer. (odd ratios = 0.87, 95% CI 0.65–1.09).

**Conclusion:**

Hypothyroidism may reduce the risk of breast cancer in the European population, and no significant correlation was observed between hypothyroidism and breast cancer risk in non-European populations. Due to the limited number of studies included, more large-scale, high-quality, long-term prospective cohort studies are needed.

## Background

As a global public health problem, breast cancer has an increasing incidence on a global scale [1]. According to the 2017 US cancer statistics, breast cancer has become the most common malignant tumour in women, with about 250,000 new cases each year, accounting for 30% of new malignant tumours in women [2]. Therefore, the identification of risk factors for breast cancer and the adoption of effective early prevention and intervention measures are of great significance for patients with breast cancer.

The physiology and pathology of the breast are closely related to the endocrine of the body [3]. As the largest endocrine organ in the human body, the thyroid gland has specific regulation effects on various hormone levels and cell growth and development in the body, which brings new enlightenment to the research of breast cancer [4–6]. In 1976, Kapdi et al. first proposed that hypothyroidism maybe increase the risk of breast cancer [7]. Since then, many scholars have studied the relationship between hypothyroidism and the risk of breast cancer. However, the relationship between the two diseases remains controversial [7–11]. Some studies have shown that hypothyroidism increases the risk of breast cancer [7–9]. Some studies have shown that hypothyroidism reduces the risk of breast cancer [10]. Besides, some studies have found no correlation between thyroid disease and breast cancer risk [11]. Therefore, whether hypothyroidism can increase the risk of breast cancer is worthy of further study.

Two meta-analyses have previously been studied for hypothyroidism and breast cancer risk [11, 12]. Based on previous research, we have included more prospective studies and Asian population studies to assess the relationship between hypothyroidism and breast cancer risk systematically. Besides, the impact of thyroid hormone replacement therapy on breast cancer risk was explored in this meta-analysis.

## Methods

### Search strategy

Relevant clinical literature was extracted by systematic retrieval of PubMed (Medline), EMBASE, Springer, Web of Science, and Cochrane Library electronic databases up to date to October 2019. Our search strategy included terms for: “thyroid dysfunction” or “hypothyroidism” or “HT” and “thyroid diseases” or “breast cancer” or “BC” or “breast neoplasms” or “mammarmy cancer” and “risk” or“incidence”. At the same time, we manually screened out the relevant potential literature in the references extracted.

### Inclusion and exclusion criteria

1. The inclusion criteria:
2. Types of studies: Published studies exploring the relationship between hypothyroidism and breast cancer risk;
3. Subject: Female;
4. Exposure factors: Primary hypothyroidism, the diagnosis needs to be based on the detection of thyroid function;
5. Outcome indicators: the occurrence of primary breast cancer.

The exclusion criteria:

1. Non-primary hypothyroidism due to other causes;
2. Non observational studies;
3. Insufficient information was provided or no full-text;
4. Unable to obtain full text or quality assessment of the literature;
5. Studies were repeated or publications overlapped.

### Data extraction and quality assessment

Two researchers separately conducted literature screening, data extraction, and literature quality evaluation, and any differences could be resolved through discussion or a third inspector. Information secured from the enrolled literature included: first author’s surname, year of publication, country of the population, sample size, follow-up time, and data on the relationship between hypothyroidism and the risk of breast cancer.

The Newcastle-Ottawa Scale (NOS) was used to assess the quality of the study from three aspects: cohort selection, cohort comparability, and outcome evaluation [13]. NOS scores of at least six were considered high-quality literature. Higher NOS scores showed higher literature quality.

### Statistical analysis

All data analysis was performed using Stata12.0 software. Meta-analysis was performed according to the PRISMA guidelines. The OR and 95%CI from included studies were treated with the combined effect size. After that, the heterogeneity test was conducted. When *P* ≥ 0.05 or I^2^ < 50% was performed, it mean that there was no apparent heterogeneity, and the fixed-effect model should be applied for a merger. When *P* < 0.05 or I^2^ ≥ 50% indicated high heterogeneity, the random-effect model was applied. Combined effect size, if OR > 1 indicates that hypothyroidism is an unfavorable factor for breast cancer. If OR < 1 is the opposite. Publication bias Begg funnel plot and Egger test linear regression test were used to research publication bias detection of the literature included. If *P*  < 0.05 indicates obvious publication bias.

## Results

### Process of study selection and description of qualified studies

**Fig. 1:**
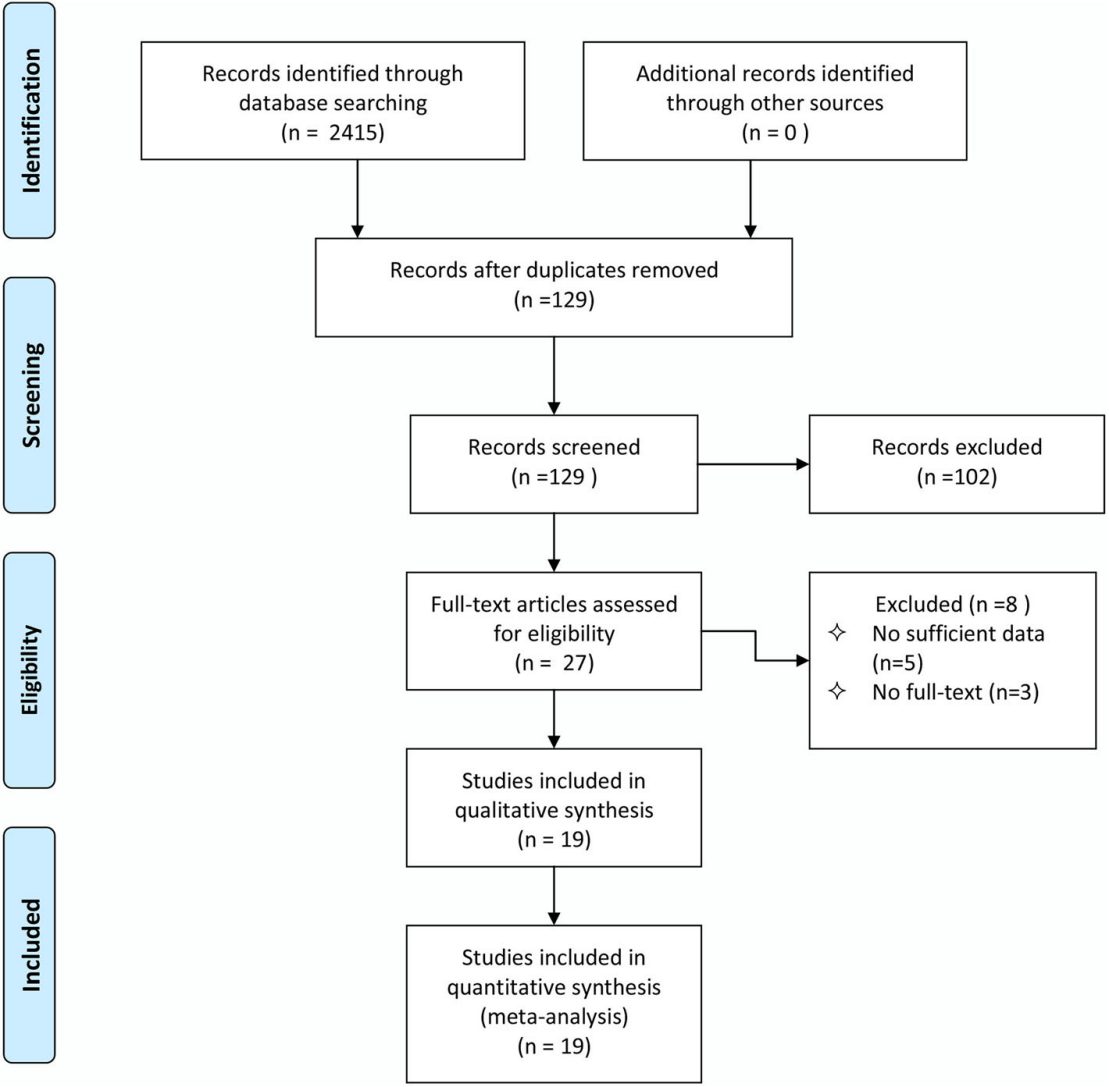
Flow chart of search strategy and study selection

A total of 2415 studies were identified on our online databases. After exclusion of duplicate references,129 articles were considered. After screening the abstract and title, 102 articles were excluded. After careful review of the full texts, 8 studies have been excluded because 5 of them did not provide relevant data, and 3 articles did not have full-text. Nineteen articles published between 1978 and 2019 met the inclusion criteria (Fig. 1).

A total of 367,416 samples from 19 studies involving were enrolled in this meta-analysis [4, 8–10, 14–28]. Six cohort studies and 13 case-control studies were included in the study. Twelve articles were studied in the European population, five in the North American population, and two in the Asian population. All articles are of high quality because of NOS score no less than 6. The chief characteristics of the enrolled materials are detailed in Table 1.

**Table 1:** Main characteristics of the included studies in our-analysis

Study	Year	Region	Sample	Median/Mean age	Follow-up	Study design	NOS
				(years)	(years)		
Adami	1978	Sweden	358	64	1	Case-control	7
Kalache	1982	UK	2352	NA	11	Case-control	6
Hoffman	1984	Sweden	1665	47.2	21.9	Cohort	8
Brinton	1984	USA	2612	NA	4	Case-control	7
Moseson	1993	Canada	1101	54	4	Case-control	7
Smyth	1996	Ireland	400	57.2 ± 1.4	1	Case-control	7
Shering	1996	Ireland	350	NA	NA	Case-control	7
Talamini	1997	Italy	5157	55	3	Case-control	7
Simon	2002	USA	9257	NA	4	Case-control	6
Turken	2003	Prague	250	63	4	Case-control	6
Kuijpens	2005	Netherlands	2775	50.5	9	Cohort	8
Cristofanilli	2005	USA	2224	51.6 ± 12.6	3	Case-control	6
Sandhu	2009	Canada	179,462	74.9 ± 7	10	Cohort	8
Hellevik	2009	Norwegian	29,691	≥20	9	Cohort	7
Ditsch	2010	Germany	130	58.6 ± 13.5	NA	Case-control	7
Grani	2012	Italy	380	59	5	Case-control	7
Søgaard	2016	Danish	61,873	71	35	Cohort	7
Weng	2018	USA	103,466	53.3	NA	Case-control	8
Kim	2019	Korea	67,416	≥40	4	Cohort	8

### Relationship between hypothyroidism and breast cancer risk

**Fig. 2:**
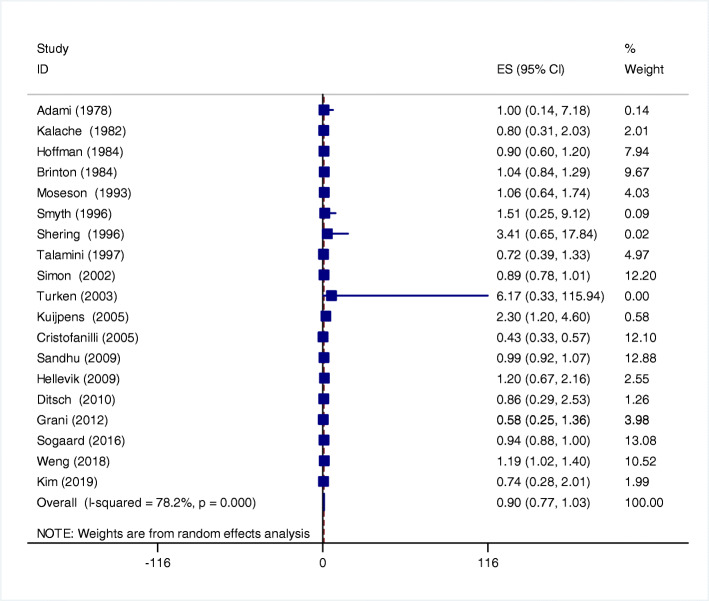
Relationship between hypothyroidism and breast cancer risk

There were 19 studies reported the relationship between hypothyroidism and breast cancer risk. With obvious heterogeneity (*I*^*2*^ = 78.2%, *p* = 0.000) among these studies, so a random effect model was used for assessment. The pooled analysis suggested that was not related to the risk of breast cancer (OR 0.90, 95% CI 0.77–1.03, *P* < 0.001)(Fig. 2).

### Subgroup analysis of hypothyroidism and risk of breast cancer

To further explore the relationship between hypothyroidism and breast cancer risk, subgroup analysis was conducted from three aspects: study type, population distribution, and follow-up time. The results of subgroup analysis were shown in Table 2. In the European subgroup, we observed that patients with hypothyroidism have a lower risk of breast cancer (OR 0.93, 95% CI 0.88–0.99, *P* < 0.001). In the subgroup with a follow-up date of more than four years, patients with hypothyroidism can reduce the risk of breast cancer, with borderline significance (OR 0.96, 95% CI 0.91–1.00, *P* < 0.001). In other subgroups, we found that hypothyroidism was not related to the risk of breast cancer.

**Table 2:** Stratiedanalysis of the relationship between hypothyroidism and breast cancer risk

Variable	No.of studies	OR(95%CI)	*P*	Heterogeneity		Model used
				*I*^*2*^	*P*_*h*_	
Region						
Europe	12	0.93 (0.88–0.99)	< 0.001	0	0.877	Fixed
North America	5	0.86 (0.60–1.11)	< 0.001	93.8%	0	Randomed
Asia	2	1.17 (0.98–1.35)	< 0.001	0	0.319	Fixed
Study design						
Case-control	13	0.85 (0.62–1.09)	< 0.001	80.4%	0	Randomed
Cohort	6	0.96 (0.91–1.01)	< 0.001	0	0.517	Fixed
Follow-up date						
> 4	7	0.96 (0.91–1.00)	< 0.001	0	0.435	Fixed
≤ 4	9	0.80 (0.54–1.07)	< 0.001	81.0%	0	Randomed

### Relationship between thyroid hormone replacement therapy and breast cancer risk

**Fig. 3:**
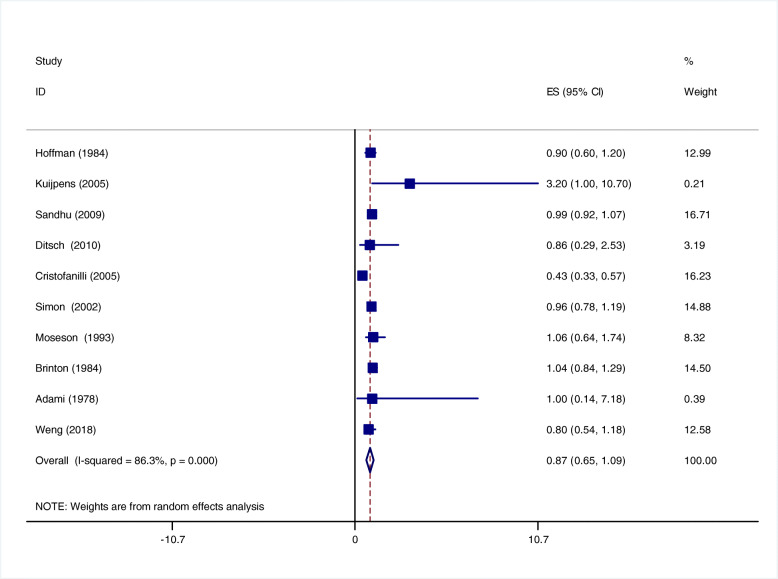
Relationship between thyroid hormone replacement therapy and breast cancer risk

A total of 10 studies reported the relationship between the use of thyroid hormone replacement therapy and the risk of breast cancer [4, 8, 9, 15, 17, 21, 23, 25, 26]. As obvious heterogeneity observed, the fixed-effect model was used(I ^*2*^ = 86.3%, *p* = 0.000). The result suggested that patients who received thyroid hormone replacement therapy was not related to the risk of breast cancer (OR = 0.87, 95% CI 0.65–1.09;*P* < 0.001) (Fig. 3).

### Publication bias

**Fig. 4:**
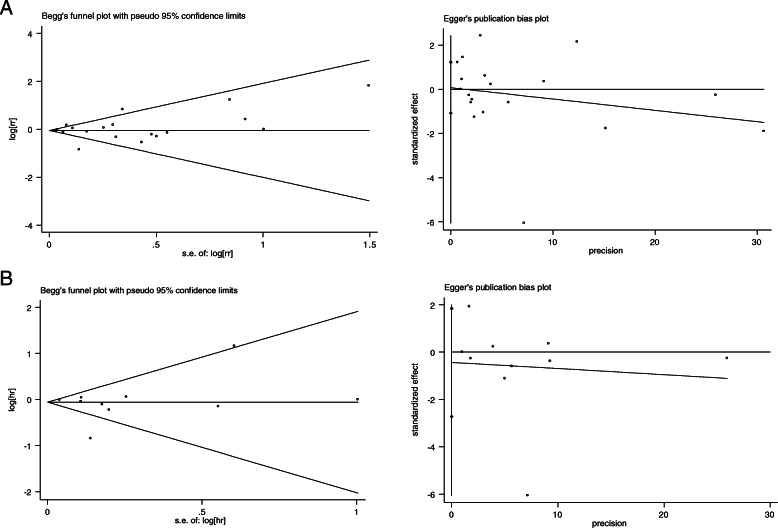
Publication bias assessment **a** hypothyroidism; **b** thyroid hormone replacement therapy

Figure 4a shows the results of publication bias for the relationship between hypothyroidism and breast cancer risk, which were evaluated by funnel plots and Eggers test. The Begg test (*Pr* = 0.529) and the Egger test(*P* = 0.892) were used to detecting publication bias showed that there was no possibility of publication bias. As shown in Fig. 4b, there were no publication biases in the 10 articles on the study of thyroid hormone replacement therapy. The Egger test was *P* = 0.672, and the Begg test was *Pr* = 0.858.

### Sensitivity analysis

**Fig. 5:**
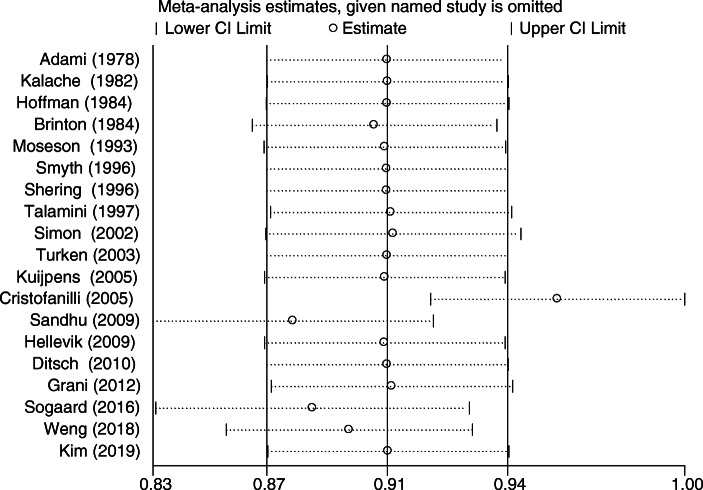
Sensitivity analysis for relationship between hypothyroidism and breast cancer risk

The results of sensitivity analysis are generally stable, and the primary source of heterogeneity is in the research of Cristofanilli et al. [23].(Fig. 5). So we excluded the literature of Cristofanilli and analyzed the other studies. The results revealed that the hypothyroidism could reduce the risk of breast cancer was borderline significant (OR:0.96 95%CI:0.92–1.00, *P* < 0.001), and there was no heterogeneity(I^2^ = 0, *P* = 0.577).

## Discussion

More than 100 years ago, Beatson et al. used thyroid extracts to treat patients with metastatic advanced breast cancer. The condition was significantly alleviated, sparking interest in exploring the relationship between thyroid and breast cancer [29]. Subsequently, a prospective study enrolled 2775 women, and 61 women with earlier diagnosis of hypothyroidism observed the occurrence of breast cancer during follow-up showed that low serum free thyroxine levels increased the risk of breast cancer [8]. In 2016, a prospective cohort study of 61,873 women with hypothyroidism and 80,343 hyperthyroidism found that hypothyroidism slightly reduced the risk of breast cancer [10]. However, a prospective cohort study of 89,731 women with autoimmune hypothyroidism and 89,731 women with normal thyroid function indicated that autoimmune hypothyroidism was not associated with breast cancer risk [25]. Besides, some animal experiments also reflect the relationship between the two [30, 31]. Animal experiments by López Fontana et al. found that hypothyroidism mice inhibit the development of breast cancer and promote the apoptosis of breast cancer cells due to the low expression of β-chain protein and activation of the apoptotic pathway on the tumour cell membrane [30]. Due to the inconsistency of the above conclusions, we performed a meta-analysis to evaluate the relationship between hypothyroidism and breast cancer risk.

A total of 19 studies were included in this meta-analysis, and the results showed that patients with hypothyroidism not related to the risk of breast cancer. However, there was significant heterogeneity among the included studies. After subgroup analysis and sensitivity analysis, we found that Cristofanilli’s research may cause heterogeneity [23]. Cristofanilli’s research is a retrospective study, and the diagnosis of hypothyroidism patients was based on the information recorded in the medical records, which may lead to the bias risk of misclassification and have a positive impact on the positive results of this study [23]. After excluding Cristofanilli’s research, we found that patients with hypothyroidism had a lower risk of breast cancer with borderline significance [23]. The results of the meta-analysis are inconsistent with the findings of Hardefeldt et al. and Angelousi et al. [11, 12]. Perhaps because our study included more prospective studies and Asian population cohort study. In addition, we evaluated the risk of breast cancer in thyroid hormone replacement therapy and show that patients who received thyroid hormone replacement therapy was not related to the risk of breast cancer.

In the analysis of the European population, the results show that hypothyroidism may reduce the risk of breast cancer. We also found that patients with hypothyroidism can reduce the risk of breast cancer was borderline significance in the subgroup with more longer follow-up date. However, the relationship between the two was not observed in North American and Asian populations. The possible reasons for these disparities may be as follows. First, follow-up time may be the main contributors to this difference. A longer follow-up is required to demonstrate the relationship between hypothyroidism and breast cancer risk. In the meta-analysis, five studies provided North American population data, and two reported Asian population data. However, only one of seven non-European studies’ follow-up time for more than 4 years. Second, the differences may be attributed to different ethnicities sharing different gene-gene and gene-environmental backgrounds. Third, social and environmental factors are another critical cause for this difference. With these in mind, our findings suggest that hypothyroidism may reduce the risk of breast cancer only in the European population and more large-scale, high-quality, long-term prospective cohort studies are still needed to study on different human populations.

The following may explain the potential relationship between hypothyroidism and the risk of breast cancer. Healthy mammary epithelial cells can express a large number of T3 receptors, and breast cancer cells have a similar ability to bind to T3 [32]. T3 has an estrogen-like effect that promotes the growth of mammary gland lobes and stimulates normal breast tissue differentiation [33, 34]. Therefore, T3 can mimic the effect of estrogen on the proliferation of breast cancer cells. When the concentration of T3 is low in vivo, it may inhibit the proliferation of breast cancer cells. Hypothyroidism may reduce the risk of breast cancer by affecting T3 concentration.

Some basic experiments support this theory. In 2002, Gonzalez-Sancho et al. studied the relationship between T3 and breast cancer [35]. It was found that there is an over-expressed T1 gene in human breast cancer cells, and T3 inhibits the proliferation of mammary epithelial cells by inhibiting the expression of cyclin D1 and T1, thereby inhibiting the proliferation of breast cancer cells [35]. After that, Martinez-Iglesias found that hypothyroidism can inhibit the growth of breast cancer cells [31]. In 2010, Tosovic conducted a prospective study of T3 levels associated with breast cancer risk and found that T3 levels in postmenopausal women were positively correlated with breast cancer risk in a dose-response manner [36]. Therefore, we suspect that hypothyroidism through lower levels of T3 could reduce the incidence of breast cancer. Our meta-analysis results also confirm the above conjecture.

However, this conclusion needs to be taken with caution, as this study has several limitations. First, the studies that have been included do not adjust for important risk factors for breast cancer. Second, in subgroup analysis, for example, there are only two articles in Asian studies, and we should be cautious about the results of Asian analysis. Third, the results of this meta-analysis indicate that there is a large heterogeneity between studies. Fourth, follow-up time at different endpoints cannot be uniform. Finally, publication bias cannot be avoided entirely.

## Conclusion

Hypothyroidism may reduce the risk of breast cancer in the European population, and no significant correlation was observed between hypothyroidism and breast cancer risk in non-European populations. Furthermore, there was no obvious correlation between thyroid hormone replacement therapy and breast cancer risk. It is necessary to conduct a large sample size, strictly controlled prospective study of hypothyroidism patients further to demonstrate the relationship between hypothyroidism and breast cancer risk.

## Data Availability

All the published articles and data were available online.

## Abbreviations

OR: Odd ratios
CI: Confidence intervals
NOS: Newcastle-Ottawa Scale
